# Primary health care beyond COVID-19: dealing with the pandemic in Cameroon

**DOI:** 10.3399/bjgpopen20X101113

**Published:** 2020-09-09

**Authors:** Lundi-Anne Omam Ngo Bibaa

**Affiliations:** 1 Assistant Executive Director, Health, Reach Out NGO, Buea, Cameroon

**Keywords:** COVID-19, Primary Health Care, New Normal, Cameroon, Delivery of health care

## Background

The World Health Organization (WHO) declared COVID-19 a global pandemic on the 11 March 2020, after over 118 000 cases were confirmed worldwide from 114 countries,^1^ and, as of 8 June 2020, 6 931 000 confirmed cases were recorded globally.^2^ Overcrowding, poverty, poor hygiene practices, and weak information management systems are named as key among other factors and social determinants of COVID-19 spread.^3^ Due to the rapid global spread of SARS-CoV-2 (the virus causing COVID-19), causing an unprecedented number of deaths, many countries around the world imposed periods of shutdowns with a resultant change in service delivery models of primary care.^4^ In high income countries, remote consultations by telephone and video were quickly rolled out as alternative models of care at primary health care (PHC) level.^5^ Since the confirmation of the first COVID-19 case in Cameroon on 6 March 2020, policymakers, scientists, public health experts, and emergency specialists have been mobilised to respond to the outbreak, seeking solutions to contain the spread of the virus.^6^ Very little attention has been paid, however, to strengthening the PHC system to contain the spread of the virus in communities. Most efforts to control COVID-19 have been limited to central and regional laboratories and hospitals,^6^ forgetting the importance of PHC in emergency preparedness, response, and recovery.^7^ The WHO recommends the involvement of PHC in triaging of patients presenting with COVID-19 symptoms while ensuring continuum of service delivery of essential health care.^8^

### Primary health care in the COVID-19 context

Cameroon has a three-level model of health system; the central, intermediate, and peripheral level. The peripheral level emulates the WHO’s PHC health district (HD) model, and comprises of 189 HDs and 5284 health areas. Care provision at PHC is at three levels with a defined set of preventive and curative services: (1) the health area level, comprised of a health centre which serves as the first point of contact; (2) the HD level with a district hospital, serving as a referral facility for the health centre; (3) the district health service that coordinates and supervises all PHC facilities. Despite this, there is no proper gatekeeping mechanism to avoid by-pass. The health centres and district hospitals serve an average of 8230 persons per facility. There are a total of 2282 clinics and 146 district hospitals nationally, headed by a state registered nurse and medical doctor respectively. The majority of doctors deployed to work in district hospitals are GPs. Nurse to patient and doctor to patient ratio stands at 1:4260 and 1:15 939 respectively. Cameroon’s PHC system is designed to bring essential health care close to the population, yet emergency preparedness, response, and recovery remain weak.^9^

Cameroon’s number of confirm COVID-19 cases stands at 10 140 cases as of 15 June 2020; the highest in Central Africa. Despite this high number of COVID-19 cases, the response is still highly centralised.^6^ The COVID-19 crisis has exposed weaknesses and gaps in many PHC systems in responding to the outbreak. Although many innovative models of care have emerged at PHC level in high income countries,^10^ fewer innovations have been seen in Africa and Cameroon in particular. There has, however, been rapid integration of Infection Prevention and Control (IPC) measures into some PHC service delivery protocols to ensure continuous provision of essential health care. An example is the introduction of a COVID-19 IPC module into the national malaria training package for community health workers. This is coupled to the rapid sensitisation on COVID-19 prevention methods by the government,^6^ and civil society organisations.

Field reports suggest that many PHC centres in Cameroon that receive and see most patients in communities do not have basic personal protective equipment (PPE), are understaffed, do not benefit from training on IPC, and have limited capacity to support surveillance and contact tracing.^11^ Further research is required by public health professionals and researchers to present the real national picture of the IPC state of health facilities in Cameroon.

This is coupled to the fact that telemedicine is not common practice in the country, especially for rural, hard to reach, and conflict-affected communities. This might be linked to the difficulty in accessing telecommunication networks in many communities. If Cameroon is to succeed in eliminating COVID-19, strengthening PHC systems to offer essential health services should be part of its ‘new normal’.

### What the ‘new normal’ should look like

Faced with this pandemic, societies keep talking of the ‘new normal’: how communities need to learn to adapt, live, and work in the COVID-19 context. Khan *et al* have suggested what the ‘new normal’ could be for PHC in a high income setting.^12^

**Figure 1.**
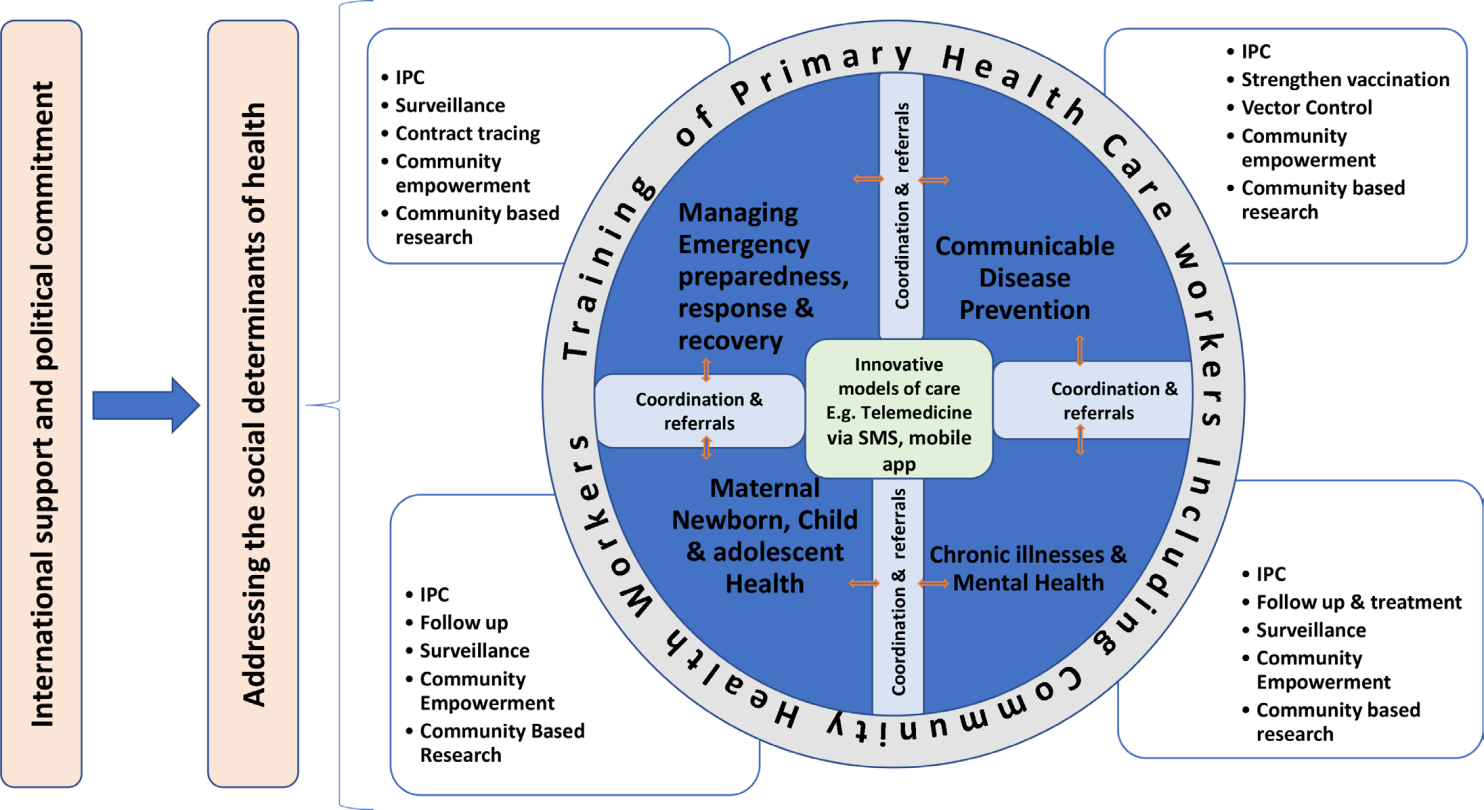
Primary Health Care Framework in the ‘new normal’ within the context of COVID-19

Thus, it will be worthwhile reflecting on how essential health care can be delivered to communities at PHC level in the ‘new normal’ within a lower-middle income country like Cameroon. Considering the importance of ensuring the continuum of primary care in preventing the high morbidity and mortality rates from communicable and non-communicable diseases — and the re-emergence of other disease outbreaks like cholera, measles, meningitis, poliomyelitis, and yellow fever — it is imperative to develop the models of PHC systems needed to operate in the ‘new normal’. A conceptual framework based on the ideology of the ‘new normal’ is designed in this article to illustrate how PHC should be in the new way of delivering health care in a low and middle-income setting like Cameroon (Figure 1).

This framework illustrates what needs to be considered by policymakers and government in strengthening PHC systems to ensure the continuous provision of care in a developing context. From the framework, it is evident that key sectors of a PHC system — (1) management of communicable diseases, (2) management of chronic and non-communicable diseases including mental health, (3) follow up of maternal, neonatal, child, and adolescent health, and (4) management of emergency preparedness response and recovery — should all be centred around innovative models of service delivery. Innovative models of care like GiftedMom and Idocta Africa, which are Cameroon-based telemedicine platforms, could be leveraged to provide PHC consultations to communities during disease outbreaks like COVID-19.

All four sectors in this framework should systematically integrate IPC measures into patient consultation, treatment, and management. Surveillance, community empowerment, and community-based research are important aspects of these PHC sectors and should be part of the ‘new normal’ of service provision at PHC level. Furthermore, the framework showcases that coordination and patient referral ought to be intertwined with effective collaboration between each sector of this framework. In all, the training of PHC practitioners including community health workers encompasses all sectors of PHC systems.

This framework also highlights that addressing the social determinants of health is important in every PHC system, especially when considering the way forward post-COVID-19. Considering the burden COVID-19 places on health systems, mobilisation of international support and a strong political commitment is required to prioritise, invest in, and strengthen all PHC sectors of this framework.

## Conclusion

The role of PHC during emergencies cannot be overemphasised. With strong PHC systems, provision of essential health care will not be discontinued or threatened during pandemics like COVID-19, and the occurrence of other disease outbreaks might be kept under control. The goal of ensuring everyone has access to health care whenever they are in need will be guaranteed, and the response against pandemics will be stronger and more cost-effective.
